# A cell size threshold triggers commitment to stomatal fate in *Arabidopsis*

**DOI:** 10.1126/sciadv.adf3497

**Published:** 2023-09-20

**Authors:** Yan Gong, Renee Dale, Hannah F. Fung, Gabriel O. Amador, Margot E. Smit, Dominique C. Bergmann

**Affiliations:** ^1^ Department of Biology, Stanford University, Stanford, CA 94305, USA.; ^2^ Donald Danforth Plant Science Center, St. Louis, MO 63132 USA.; ^3^ Department of Developmental Biology, Stanford University, Stanford, CA 94305, USA.; ^4^ Howard Hughes Medical Institute, Stanford University, Stanford, CA 94305, USA.

## Abstract

How flexible developmental programs integrate information from internal and external factors to modulate stem cell behavior is a fundamental question in developmental biology. Cells of the *Arabidopsis* stomatal lineage modify the balance of stem cell proliferation and differentiation to adjust the size and cell type composition of mature leaves. Here, we report that meristemoids, one type of stomatal lineage stem cell, trigger the transition from asymmetric self-renewing divisions to commitment and terminal differentiation by crossing a critical cell size threshold. Through computational simulation, we demonstrate that this cell size–mediated transition allows robust, yet flexible termination of stem cell proliferation, and we observe adjustments in the number of divisions before the differentiation threshold under several genetic manipulations. We experimentally evaluate several mechanisms for cell size sensing, and our data suggest that this stomatal lineage transition is dependent on a nuclear factor that is sensitive to DNA content.

## Teaser

Plant epidermal stem cells use their own size as a cue to decide whether to self-renew or differentiate.

## INTRODUCTION

**Fig. 1.**
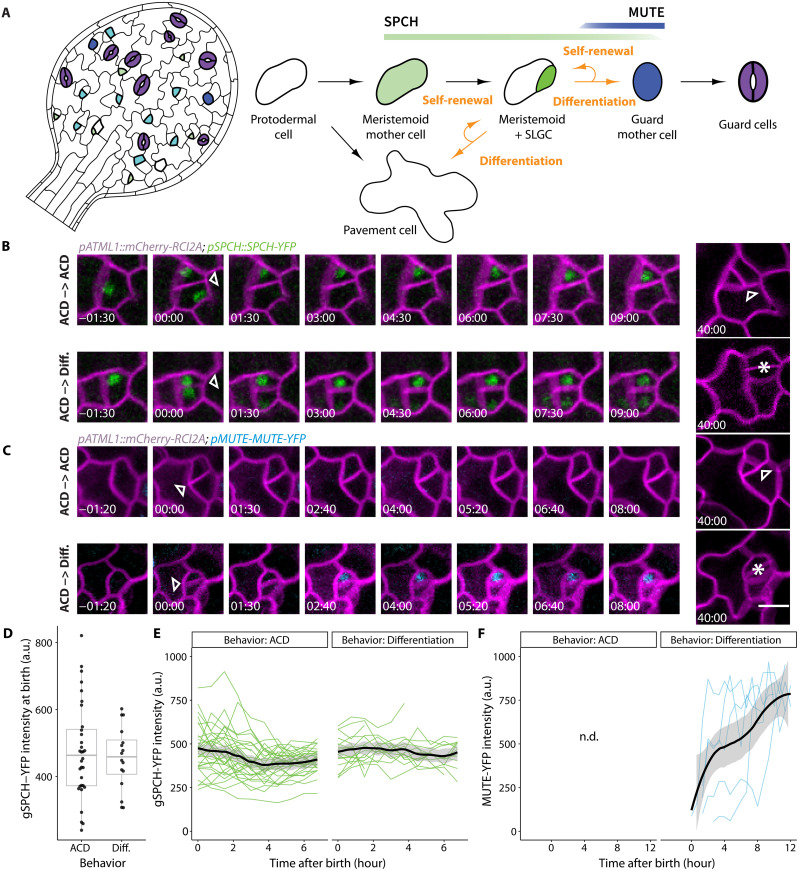
Transitions from self-renewal to differentiation in stomatal lineage meristemoids are not predicted by the expression of the transcription factors SPCH or MUTE. (**A**) Cartoon of stomatal lineage cells and lineage trajectory (right) and their spatial arrangements (left). Protodermal precursor cells undergo asymmetric “entry” divisions producing a meristemoid (green) and a stomatal lineage ground cell (SLGC; white) daughter. Meristemoids undergo additional “amplifying” asymmetric cell divisions (ACDs) before becoming guard mother cells (GMCs; blue). GMCs divide once, symmetrically into a pair of guard cells (GCs; purple). SLGCs typically become pavement cells. (**B** and **C**) Time-lapse analysis of the dynamics of *pSPCH::SPCH-YFP* [(B) green] and *pMUTE::MUTE-YFP* [(C) blue] reporters during ACDs in 3-dpg cotyledons followed by lineage tracing. SPEECHLESS (SPCH) and MUTE reporters were imaged every 40 min for 16 hours, returned to ½ Murashige and Skoog plates, and reimaged to capture the division and fates of the meristemoid daughters from ACDs. Two examples are shown for each reporter where the daughter meristemoid either undergoes another ACD or becomes a GMC which later divides into GCs. 00:00 (hours:minutes) marks cell plate formation. Cell outlines are visualized by plasma membrane reporter *pATML1::RCI2A-mCherry* (magenta). Arrowheads and asterisks indicate asymmetric meristemoid and symmetric GMC divisions, respectively. (**D**) Quantification of SPCH-YFP reporter levels at birth for meristemoids that will either undergo additional ACDs or differentiate and become stomata. (**E** and **F**) Quantification of SPCH-YFP reporter (E) or MUTE-YFP reporter (F) levels and dynamics in cells with either behavior. Trends with 95% confidence intervals are shown as black lines with gray bands. Sample sizes: (D and E) >16 cells per genotype, (F) 5 cells. Scale bar, 10 μm. a.u. arbitrary units; n.d. not detected.

During development, stem cells balance competing needs for proliferation and differentiation to control the final size and cellular composition of tissues. In systems that experience unpredictable or variable conditions, such as the gut epithelium (*1*) and muscle satellite cell (*2*, *3*) lineages, a flexible developmental program requires that this balance be dynamically altered in response to internal or external cues. The *Arabidopsis* stomatal lineage is a model for the study of such flexible developmental programs (*4*). During leaf growth, stem cells of the stomatal lineage—meristemoids and stomatal lineage ground cells (SLGCs)—undergo a variable number of self-renewing asymmetric cell divisions (ACDs) before committing to terminal differentiation as stomata or pavement cells, respectively (Fig. 1A). Notably, both meristemoid and SLGC proliferation can be tuned by hormonal, nutrient, and environmental inputs (*5*–*12*). This flexible developmental program is deeply conserved across land plants and functions to optimize mature leaf physiology to an individual’s unique local environment (*13*, *14*).

A group of closely related basic helix-loop-helix (bHLH) transcription factors controls stem cell behavior in the stomatal lineage (*15*–*18*). Among these transcription factors, SPEECHLESS (SPCH) initiates the lineage and is required for continued proliferation of both meristemoids and SLGCs (*16*, *19*), while MUTE, a direct target of SPCH, is activated in meristemoids after one to three asymmetric divisions and triggers guard mother cell (GMC) and, subsequently, stomatal differentiation (*17*, *20*). Whereas SPCH is necessary for the eventual activation of MUTE, the factors that determine the timing of the SPCH to MUTE transition, and thus of terminal differentiation into stomata, are currently unknown.

Here, we show that commitment to stomatal fate is triggered by crossing a cell size threshold. Repeated asymmetric division of meristemoids decreases cell size until the critical threshold is reached. Genetic manipulations of initial meristemoid size and division asymmetry directly affect the timing of the switch between self-renewing proliferation and terminal commitment. Modeling stem cell behavior shows that imposing a cell size threshold for differentiation is sufficient to accurately predict the number of asymmetric divisions stem cells undergo before transitioning and suggests a mechanism by which cell size may integrate environmental information to control the number of meristemoid divisions (also known as amplifying divisions) in developing leaves. Molecular mechanisms known to sense cell size rely on scaling relationships, either in protein levels (*21*) or related to geometry (*22*). Our experimental evaluation of these existing paradigms suggests that, in the stomatal lineage, size is sensed in the nucleus via a mechanism that is sensitive to DNA content.

## RESULTS

### SPCH and MUTE do not drive the transition from proliferation to differentiation in meristemoids

Multiple external and internal factors have been reported to affect the balance of stem cell proliferation and differentiation in the *Arabidopsis* stomatal lineage. Some of these factors influence the lineage by modulating SPCH levels (*5*, *6*, *10*), prompting us to investigate whether SPCH levels alone are sufficient to predict whether a meristemoid will proliferate or differentiate.

To monitor SPCH protein dynamics, we captured time-lapse images of the epidermis of a 3-day post-germination (dpg) cotyledon expressing a SPCHtranslational reporter (*spch-3 SPCHp::gSPCH-YFP*). We found that SPCH levels shortly after birth were poor predictors of future meristemoid behavior (Fig. 1, B, D, and E). Meristemoids expressed high levels of SPCH after birth, irrespective of fate (Fig. 1, D to E). In contrast, MUTE protein levels were highly correlated with meristemoid behavior, appearing exclusively in meristemoids that would differentiate several hours later (Fig. 1, C and F). This is consistent with MUTE’s role in establishing GMC identity (*20*) but also suggests that *MUTE* expression is a consequence, rather than a cause, of the decision to differentiate.

With the resolution made possible by single-cell RNA sequencing (scRNA-seq), we also investigated whether subclasses of meristemoids could be distinguished by considering SPCH in combination with other suites of genes. Although several independent scRNA-seq studies exquisitely resolved a unidirectional trajectory from *MUTE* expression to stomatal guard cell differentiation (*19*, *23*), none identified distinct groups of *SPCH*-expressing cells that could be mapped onto self-renewing or differentiating behaviors.

### A cell size threshold directs the M-GMC transition

To identify factors that might influence the decision to differentiate, we surveyed the literature for correlates of meristemoid behavior. Notably, Robinson *et al*. (*24*) reported that meristemoid size declines with successive asymmetric divisions, due to a combination of division asymmetry and minimal growth between divisions, and we confirmed these findings in our data (figs. S1 and S2, A and B). This observation, coupled with the fact that most meristemoids divide at least once before differentiating (*25*), implies that cells that divide are larger than cells that differentiate.

**Fig. 2.**
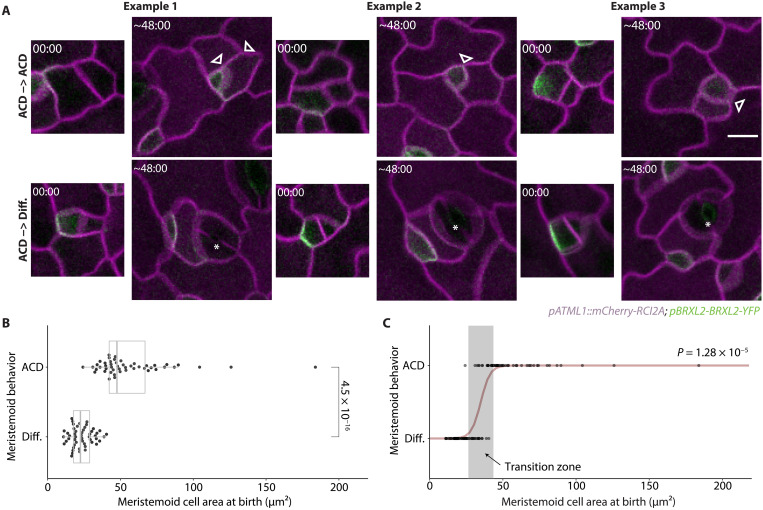
The M-GMC transition of the stomatal lineage is correlated with small meristemoid cell size at birth. (**A**) Confocal images of meristemoids at birth and the subsequent division behaviors of these meristemoids captured by lineage tracing in 3-dpg cotyledons. Meristemoids are divided into two groups based on their subsequent division behaviors: those that undergo additional ACDs or those that differentiate and become stomata. Three examples are shown for each group. Cell outlines are visualized by plasma membrane reporter *pATML1::RCI2A-mCherry* (magenta). The cell polarity reporter *pBRXL2::BRXL2-YFP* (green) was included to define cell division type. 00:00 (hours:minutes) marks cell plate formation. Arrowheads and asterisks indicate ACDs of meristemoids and GMC divisions, respectively. (**B**) Comparison of areal cell size at birth between meristemoids that acquire different fates. (**C**) Logistic regression of meristemoid behaviors based on their cell size at birth. The cell size of each meristemoid is shown as a single dot and the computed regression model is shown in dark red. The predicted transition zone where the meristemoid is predicted to have a 10 to 90% probability of undergoing another ACD is shown in a gray box. The *P* value in (B) is calculated by the Mann-Whitney test, and the *P* value in (C) is calculated by the glm.fit function with a binomial model in R (*60*). Sample sizes: (B and C) 50 cells per behavior. Scale bar, 10 μm.

To investigate whether size predicts meristemoid behavior, we quantified the birth sizes of meristemoids and recorded their subsequent behaviors (figs. S1 and S2, A and B). Birth size was operationalized as the cross-sectional area at birth, which correlates strongly with volume (*26*, *27*). Upon fitting the data to logistic regression, we found that birth size was highly predictive of meristemoid behavior (average classification accuracy: 91% ± 4.4%; see Materials and Methods): Smaller meristemoids were likely to differentiate, whereas larger meristemoids were likely to self-renew (Fig. 2C). We defined the transition size as the size at which half of the meristemoids were expected to self-renew (32 μm^2^ in 3-dpg diploid Col-0 cotyledons).

To determine whether this relationship between size and behavior is a general feature of meristemoid differentiation, we examined the distantly related eudicot tomato (*Solanum lycopersicum*). We observed a similar size bias between self-renewing and differentiating meristemoids (fig. S3), suggesting that size-restricted stomatal differentiation is not a behavior exclusive to *Arabidopsis*. In tomatoes, meristemoids can also undergo nonstomatal differentiation into pavement cells (fig. S3A) (*28*). As there is no size bias in this differentiation pathway (fig. S3B), size control appears to be a specific feature of stomatal differentiation.

### A lineage decision tree model with size as the sole determinant is sufficient to explain meristemoid behavior

**Fig. 3.**
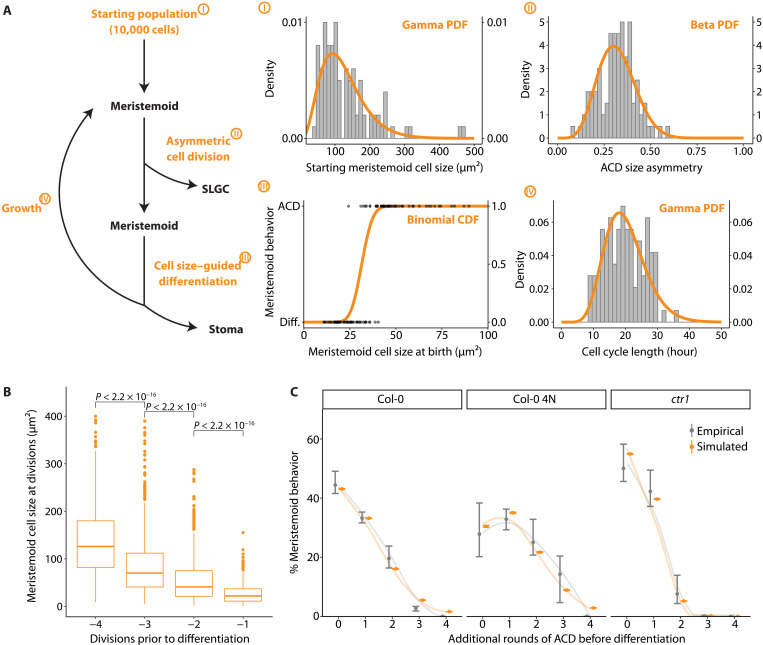
Cell size–guided M-GMC transition is sufficient to explain the self-renewal and differentiation behavior of meristemoids in silico. (**A**) Workflow of the meristemoid division tree model (left) and its key parameters (I to IV, right). A total of 10,000 meristemoid sizes are randomly drawn from a gamma distribution (I). These meristemoids divide asymmetrically, with size asymmetry drawn from a beta distribution (II). The newly formed meristemoid daughter is passed on to a cell size–guided differentiation model (III), where it differentiates with some probability based on the current size, while the SLGC is discarded. Differentiated meristemoids leave the model; the rest grow with a 3% growth rate (per hour) and a cell cycle length drawn from a gamma distribution (IV). After growth, these meristemoids are looped back to divide asymmetrically and pass through the rest of the model until all differentiate. (I) Histogram of measured starting meristemoid cell sizes (gray, *n* = 132 cells) and fitted distribution (orange). (II) Histogram of measured ACD size asymmetry (gray) and fitted distribution (orange). (III) Dot plot of measured meristemoid cell sizes at birth separated by their fates (gray) and fitted distribution (orange). (IV) Histogram of measured cell cycle lengths (gray) and fitted distribution (orange). (**B** and **C**) Outputs of the meristemoid division tree model. (B) Computed meristemoid cell sizes at ACDs before differentiation. (C) Comparison of empirical and simulated meristemoid division-differentiation behaviors. Empirical data are taken from lineage tracing experiments where behaviors of cells in the abaxial cotyledon of corresponding genotypes are tracked from 3 to 5 dpg (*8*). Smoothed conditional means are shown as lines. All *P* values are calculated by the Mann-Whitney test. Sample sizes: (A-I) 132 cells, (A-II) 98 cells, (A-III) 98 cells, (A-IV) 112 cells, (B) 913 cells, (C) >193 cells per replicate per genotype.

Our data suggest that there is a size threshold below which meristemoids are likely to differentiate. To test whether a size threshold alone is sufficient to explain observed meristemoid behaviors, we specified a stochastic and asynchronous rule-based lineage decision tree model, with size as the sole determinant of meristemoid differentiation (Fig. 3A, left). In this model, 10,000 meristemoids enter the stomatal lineage, each with a size randomly drawn from an empirically derived starting size distribution. These meristemoids divide asymmetrically, producing a meristemoid and an SLGC. The SLGCs are removed from the model, while the meristemoid undergoes a size-guided differentiation program in which smaller meristemoids have a higher chance of differentiating and exiting the model. The remaining undifferentiated meristemoids continue to grow, asymmetrically divide, and/or differentiate until all meristemoids have differentiated. The following parameters were estimated from cotyledons imaged from 3 to 5 dpg: meristemoid birth sizes, division asymmetry, the probability of meristemoid differentiation given its birth size, and cell cycle duration (Fig. 3A, right).

The model outputs included meristemoid size before and after each round of ACD, the number of meristemoids that differentiated after each round of ACD, and meristemoid size at differentiation. We compared these outputs to empirical data from our time-lapse analyses (Fig. 3, B and C) and to previously reported studies (*24*). Consistent with the work of Robinson *et al*. (*24*) and our empirical estimates (fig. S2B), meristemoid size declined with each successive division: Meristemoid daughters were, on average, 33% smaller than their parents (Fig. 3B). In addition, simulated meristemoids differentiated at an average size of 22 μm^2^, which is similar to the empirical estimate of 23.2 μm^2^ (fig. S4A). In contrast, a model where the probability of differentiation is a product of time alone fails to recapitulate the average birth size of differentiating meristemoids (fig. S4A; see Materials and Methods). We tested the sensitivity of the model to input parameters and observed that initial size, growth rate, and size of differentiation have strong effects on the number of divisions before differentiation (fig. S4, B to E). These simulations show that a differentiation program accounting for birth size alone is sufficient to recapitulate the division and differentiation behaviors of wild-type meristemoids (Fig. 3C) (*8*).

### Manipulating cell size affects the proliferative capacity of meristemoids

**Fig. 4.**
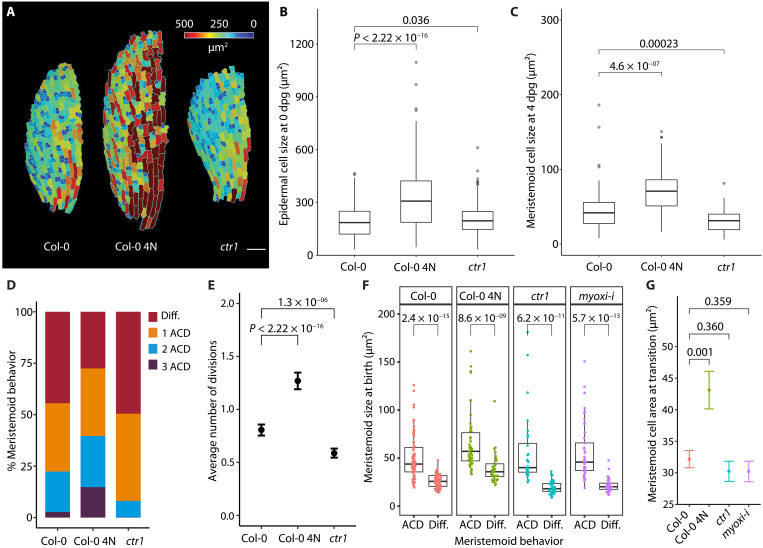
Alteration of the meristemoid size and ACD asymmetry affects a number of successive meristemoid ACDs but not the cell size of the M-GMC transition. (**A** to **C**) Comparison of cell size for leaf epidermal cells in wild-type Col-0, tetraploid Col-0 (Col-0 4N), and the *ctr1* mutant at different stages of development. (A) False-colored confocal images of the abaxial epidermis of 0-dpg cotyledons from different genetic backgrounds. mPS-PI staining images of half of the cotyledons were segmented and false-colored based on cell size in MorphoGraphX (*55*). (B) Cell size distribution of epidermal cells in Col-0, Col-0 4N, and *ctr1* cotyledons at 0 dpg. (C) Cell size distribution of meristemoids in Col-0, Col-0 4N, and *ctr1* cotyledons at 4 dpg. Meristemoids were selected from confocal images of 4-dpg cotyledons (labeled with the plasma membrane reporter *pATML1::RCI2A-mCherry*) with their cell size (surface area) measured in Fiji (*53*). (**D** and **E**) Division-differentiation behavior of meristemoid population from 3 to 5dpg, shown as the distribution (D) or as its mean, counting differentiation as zero (E). Data of Col-0 are adapted from (*8*). (**F**) Comparison of cell size at birth between meristemoids that acquire different fates in Col-0, Col-0 4N, *ctr1*, and *myoxi-i* lines. (**G**) Comparison of transition sizes computed from the data in (F). The Col-0 data are taken from Fig. 2B. All *P* values are calculated by the Mann-Whitney test. Sample sizes: (B) >500 cells per genotype, (C) >50 cells per genotype, (D and E) >636 cells per genotype, (F) >50 cells per genotype. Scale bar, 10 μm.

Previously, we identified *CONSTITUTIVE TRIPLE RESPONSE1* (*CTR1)* as a positive regulator of meristemoid proliferation (*8*), although the detailed mechanism underlying this effect was unknown. Notably, Vaseva *et al*. (*29*) reported that epidermal cell expansion was inhibited in the *ctr1* mutant, leading us to investigate whether the lack of cell expansion and consequently, a reduction in cell size, could trigger premature differentiation. While *ctr1* epidermal cells were the same size as wild-type cells at germination (0 dpg; Fig. 4, A and B), *ctr1* meristemoids were significantly smaller at 4 dpg (Fig. 4C), due to lower expansion rates (~2% versus ~3% per hour). *ctr1* meristemoids underwent fewer ACDs before differentiating (Fig. 4D), suggesting that meristemoids actively sense their size and adjust their behavior accordingly.

**Fig. 5.**
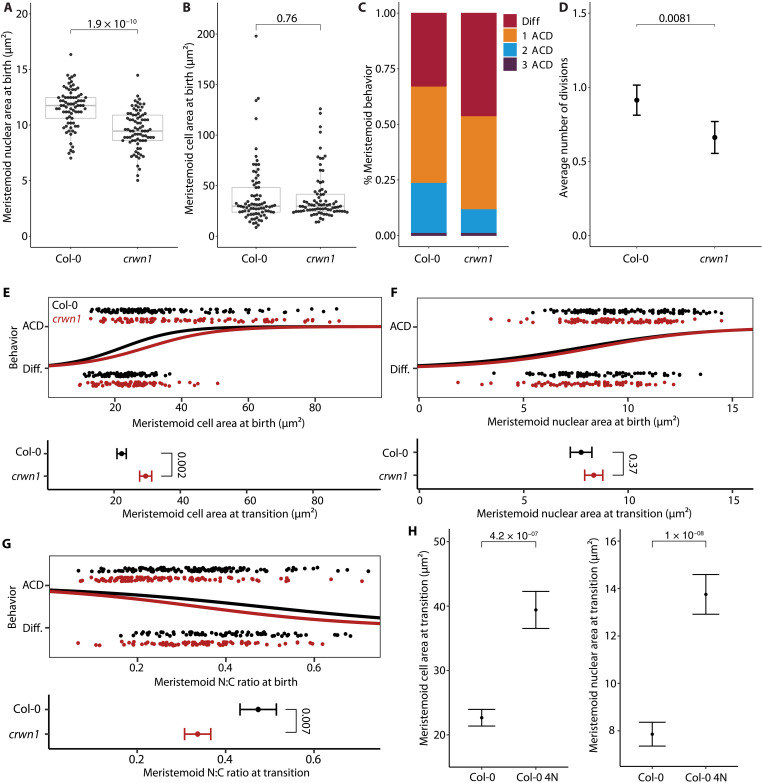
Cell size is sensed via chromatin content in the nucleoplasm. (**A** and **B**) Comparisons of nuclear area (A) and cell area (B) for wild-type Col-0 and crwn1 plants. (**C** and **D**) Comparison of a number of amplifying divisions for Col-0 and crwn1 plants, as the distribution (C) or its mean (D). (**E** to **G**) Logistic regressions of cell area (E), nuclear area (F), or N:C ratio (**G**) against meristemoid behavior, showing that relative to Col-0 meristemoids, crwn1 meristemoids transition to differentiation at the same nuclear size, but a different overall cell size and N:C ratio. Below each logistic, transition size estimates are compared. (**H**) Cell and nuclear areas of diploid and tetraploid meristemoids at transition showing that chromatin content influences both transition sizes. All *P* values are calculated by the Mann-Whitney test, except in (E) to (H), where *t* tests were performed on the estimates of transition sizes (see Materials and Methods). Sample sizes: (A) >77 cells per genotype, (B) >79 cells per genotype, (C and D) 600 cells per genotype, (E to G) >200 cells per genotype, (H) ≥100 cells per genotype.

If decreasing cell size causes premature differentiation, then increasing cell size should increase meristemoid proliferative capacity. Typical experiments to increase cell size involve genetic or pharmacological treatments that delay the G1/S transition (*21*). The stomatal lineages are asynchronous and embedded in a developing tissue and our fate assay requires that cells divide (asymmetrically as meristemoid or symmetrically as GMCs) so these manipulations are not appropriate. An alternative way to increase cell size is to use tetraploid plants (*30*). Quantification of cell size from still confocal images of a previously characterized induced Col-0 tetraploid line (*27*) revealed that both leaf epidermal cells (Fig. 4, A and B) and meristemoids (Fig. 4C) were significantly larger in the tetraploid than in diploid Col-0. We found that the meristemoid size was accompanied by an increase in the number of divisions before differentiation (Fig. 4, D and E), supporting a conclusion that cell size is linked to stomatal fate choices. A complication is that tetraploids also increase the DNA content per cell, and we cannot rule out that cells are sensing some aspect of this ploidy relationship, an issue that we return to in our final experiments (Fig. 5). However, size remains predictive of meristemoid behavior when genetic manipulations are used to alter cell size, as in *ctr1* or tetraploids, or when division asymmetry is reduced (as in *myosin-xi*; Fig. 4, F and G) (*31*). When the size-based lineage decision tree model is used to simulate meristemoid behaviors in the *ctr1* and tetraploid backgrounds, with genotype-specific parameters derived for initial cell size, growth rate, and transition size, it is again consistent with empirical data (Fig. 3C).

As the stomatal size is tightly linked to transpirative capacity (*32*), we hypothesized that size-gated differentiation of stomatal precursors may set the mature size of stomata. However, in a transgenic line where meristemoids differentiate at unusually small sizes, mature stomata are not, on average, smaller nor do we observe especially small stomata (fig. S5). These data are consistent with additional layers of size control regulating guard cell growth during their 20-fold expansion from a birth size of ~25 μm^2^ to a stomatal size of ~500 μm^2^ at maturity.

### Cells measure nuclear size to trigger differentiation

Several stem cell lineages sense cell size to inform choices about cell cycle progression, division, and differentiation. For example, in the *Caenorhabditis elegans* embryo, the germline (P) lineage undergoes four consecutive asymmetric divisions before switching to symmetric division at a threshold size where cells are too small to be able to completely segregate a polarly localized PAR protein complex to a single daughter (*22*). We tested whether a similar mechanism may sense size in the leaf epidermis. As in *C. elegans*, polar crescents (here containing BRXL2 proteins) occupy a larger fraction of the cortex in small cells than in large cells, approaching ~40% of the total circumference of small meristemoids fated to differentiate (fig. S6A). Expanding the size of the polar crescent, by expressing a *pBASL::BRX-CFP* transgene, would be expected to cause cells to differentiate at a larger size; however, we find that cells actually differentiate at slightly smaller sizes upon this manipulation (fig. S6, B to E). A caveat of this experiment is that manipulating polarity could have pleiotropic effects on cell fate; however, we can conclude that if stomatal lineage cells use cortical polarity as a cue for size-dependent differentiation, the mechanism is not the same as that described for the *C. elegans* P cell lineage.

A polarity-independent mechanism for cell size sensing was recently described in the shoot apical meristem, where cells inherit a fixed amount of the cell cycle progression inhibitor KRP4 (KIP-RELATED PROTEIN 4) in proportion to their ploidy and read out the concentration of KRP4 in nuclei of varying sizes (*33*). As nuclear size is strongly correlated to overall cell size, this “chromatin ruler” model allows cells to delay cell cycle progression until growing to a target size. To determine whether fate choices are made by meristemoids sensing their cell or nuclear size, we sought a genetic manipulation that would change nuclear size without affecting cell size. The loss-of-function *crwn1*-*1* allele (*34*) satisfies these criteria. *CRWN1* encodes a plant lamin-like protein that is involved in building the meshwork structure of the nuclear lamina (*35*). Plants homozygous for the *crwn1*-*1* allele harbor smaller nuclei at birth [*t*(154) = 6.93, *P* = 1.9 × 10^–10^; Fig. 5A and fig. S7A], but their meristemoids remain wild-type–sized (*W* = 3069.5, *Z* = −0.31, *P* = 0.76; Fig. 5B). Nuclear and cell areas remain positively correlated in *crwn1* mutants [*r* = 0.63, *t* (48) = 5.6, *P* = 9.29 × 10^–7^; fig. S7, C and D). The loss of *CRWN1* alone is not associated with major changes in gene expression or chromatin accessibility, though multiple mutants with related lamin-like proteins can affect these processes (*36*).

If cells were sensing overall size, then we would expect the population of *crwn1* meristemoids to undergo the same number of amplifying divisions as wild-type meristemoids; if, however, cells were sensing nuclear size, then *crwn1* meristemoids should undergo fewer rounds of amplifying divisions. In other words, they should differentiate at larger cell sizes, but similar nuclear sizes. Lineage tracing experiments of *crwn1* cotyledons (comprising 3- and 5-dpg time points) revealed a significant decrease in the number of amplifying divisions per meristemoid (mixed effects model: β = 0.25 ± 0.060, *P* = 0.0019; Fig. 5, C and D). Through time-lapse imaging, we found that *crwn1* meristemoids differentiate at larger cell sizes (*P* = 0.002; Fig. 5E), but similar nuclear sizes (*P* = 0.37; Fig. 5F), indicating that nuclear size is the key factor. Consistent with this, *ctr1* meristemoids, which we showed transition earlier than those in Col-0 (Fig. 4, D and E), have smaller nuclei than Col-0 (fig. S8).

Because the nuclear-to-cell area ratio (N:C ratio) contributes to developmental transitions in other systems (*37*), we also compared the N:C ratios at transition and found that wild-type and *crwn1* meristemoids transition at different N:C ratios (*P* = 0.007; Fig. 5G). Finding similar cell sizes but smaller nuclear sizes in *crwn1* meristemoids also lets us consider whether cells might sense cytoplasmic volumes. If there were a cytoplasmic sensor, then we would expect differentiation to be triggered at lower overall cell sizes in *crwn1*. However, *crwn1* meristemoids instead differentiate at higher overall cell sizes (Fig. 5E). Together, these data are most consistent with a nuclear size sensor.

The significant difference in transition size between Col-0 2N and Col-0 4N (Fig. 4G) hints that the sensor may respond to DNA content, similar to how KRP4 acts as a size sensor in the shoot meristem (*33*). We revisited tetraploids, this time tracking previously generated Col-0 lines expressing both plasma membrane and nuclear markers (*27*) to enable us to capture cell and nuclear sizes in the same meristemoids during development. Upon quantifying transition sizes, we found that tetraploid meristemoids transition at larger nuclear and larger overall cell sizes than diploid Col-0 meristemoids bearing the same markers (Fig. 5H). Together, these data suggest that meristemoids sense size through nuclear factors engaging with chromatin, raising the question of whether KRP4 could be the size sensor in our system. While the concentration of KRP4 could likewise be elevated in small meristemoids, KRP4 is unlikely to trigger stomatal differentiation directly, given its function as a cell cycle inhibitor and the observation that both meristemoids and GMCs continue to divide.

## DISCUSSION

In this study, we show that commitment to stomatal fate is triggered when meristemoid size falls below a critical threshold. Genetic manipulations of meristemoid size are accompanied by changes in proliferative capacity, indicating that meristemoids actively sense their size, likely through a nuclear factor.

We draw inspiration from previous work on homeostatic populations of stem cells that use size to control the timing of division (*33*, *38*), often via dilution of a cell cycle progression inhibitor. In contrast to these models, however, meristemoids use size to control the timing of differentiation, not division, and do not display long-term size homeostasis, instead shrinking markedly over successive divisions to reach the differentiation threshold. This system is consistent with a model in which a differentiation factor becomes concentrated over successive divisions. While the identity of this factor is unknown, our work has uncovered several of its features. On the basis of results from the *crwn1* and tetraploid analyses, we propose that size is sensed through a nuclear factor that is sensitive, but not necessarily bound, to chromatin. As meristemoid size declines, the factor becomes sufficiently concentrated and activates the expression of GMC-specific genes, including *MUTE*, to drive terminal differentiation.

Meristemoid shrinkage requires uncoupling cell growth from division. In other shrinking stem cell lineages, such as *Drosophila* type II neuroblasts, shrinkage requires extensive metabolic remodeling during asymmetric division (*39*), including changes in the expression of core oxidative phosphorylation enzymes. It is tempting to speculate that the slow growth rates observed in meristemoids are caused by similar metabolic remodeling. Notably, a recent report (*40*) showed that meristemoids have elevated levels of hydrogen peroxide (H_2_O_2_), partly due to SPCH-driven repression of H_2_O_2_-scavenging enzymes CAT2 (CATALASE 2) and APX1 (ASCORBATE PEROXIDASE 1). As hydrogen peroxide is also a byproduct of oxidative phosphorylation (*41*), future work should explore a potential link between oxidative phosphorylation and stomatal development.

Manipulations of starting meristemoid size or growth rates (in tetraploid and *ctr1* plants, respectively) affect the number of amplifying divisions and thus the stomatal index of mature leaves (Fig. 4). In general, coupling cell size to differentiation may provide a quantitative tuning point to integrate internal and external signals that control meristemoid self-renewal. Cell size may be an especially attractive integrator for several reasons. First, in principle, meristemoid shrinkage can be tuned to a wide range of values, allowing flexible control of the number of divisions before differentiation. Second, many environmental and hormonal inputs are known to influence cell size in plants by controlling the rate of cell expansion and/or cell cycle length (*29*, *42*, *43*). Last, the evolution of tissue- and stage-specific cell cycle regulators (*44*, *45*) may allow organisms to fine-tune cell size in specific cell lineages or developmental stages.

This work provides a quantitative assessment of the contribution of cell size, and in particular nuclear size, to fate transition. It does not, however, identify the precise molecules or mechanisms responsible for sensing the critical size threshold. We are also limited by the genetic or pharmacological tools available to make discrete manipulations of cell size without disrupting cell cycle progression or generating pleiotropic effects on the development of this multicellular organism. Our work identifies a situation that is not a perfect match for currently available models of cell size sensing, yet the biological phenomenon of flexible and tunable size-dependent fate transition is widespread in nature.

Over the years, cell biologists have made substantial inroads on the question of why cell size matters. A growing body of work has shown that size matters for division competence (*21*), biosynthetic capacity (*46*), metabolic flux (*39*), and stem cell exhaustion (*47*). Our study adds fate specification to the compendium of size-regulated processes, building on previous work in *C. elegans* (*22*) and *Volvox carteri* (*48*) germ cell fate specification and, in somatic stem cell lineages, in *Drosophila* (*39*). Future work to identify the specific molecules may reveal cell type–specific programs, and here, it will be interesting to see if homologous or analogous molecules mediate other size-dependent developmental transitions. The system also has the potential to reveal general principles about using cell size as the cue for differentiation and whether a single mechanism coordinates both sensing and responding to cell size, or whether the size sensor is separable from the mechanisms that drive differentiation.

## MATERIALS AND METHODS

### Plant material and growth conditions

All *Arabidopsis* lines used in this study are in the Col-0 background, and wild type refers to this ecotype. *Arabidopsis* seeds were surface-sterilized by bleach or 75% ethanol and stratified for 2 days. After stratification, seedlings were vertically grown on ½ Murashige and Skoog (MS) media with 1% agar for 3 to 14 days under long-day conditions (16-hour light/8-hour dark at 22°C) and moderate intensity full-spectrum light (110 μE).

Previously reported mutants and transgenic lines include the following: *pSPCH::SPCH-YFP pATML1::mCherry-RCI2A* in *spch-3* (*19*), *pMUTE::MUTE-YFP pATML1::mCherry-RCI2A* (*49*), *pBRXL2::BRXL2-YFP pATML1::mCherry-RCI2A* (*50*, *51*), *pBRXL2::BRXL2-YFP pATML1::mCherry-RCI2A* in *ctr1* (*8*), *p35S::PIP2A-RFP* in *basl-2* (*50*), *pBRXL2::BRXL2-YFP pATML1::mCherry-RCI2A* in *myoxi-i* (*31*), *pATML1::mCherry-RCI2A pBASL::BRX-CFP* (*50*), *pATML1::H2B-mTFP pATML1::mCit-RCI2A* (*27*), and tetraploid Col-0 *pATML1::H2B-mTFP pATML1::mCit-RCI2A* (*27*). We created *crwn1*-*1 pATML1::H2B-mTFP pATML1::mCit-RCI2A* by crossing *crwn1*-*1* (*34*) with *pATML1::H2B-mTFP pATML1::mCit-RCI2A* (*27*) and tetraploid Col-0 *pATML1::mCherry-RCI2A* by transforming ABRC stock number CS922919 with the *pATML1::mCherry-RCI2A* construct.

### Microscopy, image acquisition, and image analysis

All fluorescence imaging, time-lapse, and time-course experiments were performed as described in (*52*). Measurements “taken at birth” refer to data captured from the first frame after the appearance of the newly formed cell plate, which is <40 min from time-lapse images. For additional replicates of data presented in Fig. 5 (E to H), a modified time-lapse protocol was used to capture birth sizes of meristemoids while enabling higher throughput of multiple genotypes and samples. Briefly, seedlings were imaged at 0, 3, and 6 hours, and then removed from the imaging slide and returned to MS agar plates and grown under standard light and temperatures. To track cell fates, the same seedlings were reimaged 48 hours later. As meristemoids grow slowly (~3% per hour), this modified sampling strategy is only expected to increase birth size measurement error due to cell growth from ~1 to ~5%. To quantify SPCH protein levels (Fig. 1D), we captured a time lapse of a 3-dpg cotyledon expressing the translational reporter *pSPCH::gSPCH-YFP.* We randomly selected meristemoids that were born during this time lapse and recorded their subsequent behaviors (self-renewal versus differentiation). Mean SPCH-YFP intensities were quantified as the raw integrated density of a summed projection divided by the area of the region of interest in square micrometers. Similarly, MUTE protein levels (Fig. 1E) were measured from a time lapse of a 3-dpg cotyledon expressing *pMUTE::MUTE-YFP.* We segmented, tracked, and measured the mean fluorescence intensity of MUTE-YFP using the TrackMate Fiji plugin (*53*).

To quantify epidermal cell size at 0 dpg (Fig. 4, A and B), mature embryos were dissected from seeds and stained with mPS-PI (modified pseudo-Schiff propidium iodide) staining as described previously (*54*). The stained embryos were imaged using a Leica SP8 confocal microscope and MorphoGraphX (*55*) was used to create a surface mesh containing the epidermal signal that was then segmented to quantify cell surface areas. In Figs. 2 to 4, the meristemoid cell area was measured using the polygon tool in Fiji (description of the process in fig. S1). In Fig. 5, nuclear and plasma membrane signals were segmented semiautomatically using ilastik (*56*) with examples of source and output images provided in fig. S1B; nuclear and cell areas were quantified in Fiji (*53*). A test of the relationship between nuclear area and nuclear volume is provided in fig. S7B. In fig. S6A, crescent sizes were measured using POME (polarity measurement) (*51*). In fig. S8, to quantify nuclear size in the absence of genetically encoded nuclear markers, Col-0 and *ctr1* nuclei were stained using a protocol adapted from (*57*). Briefly, 5-dpg seedlings were fixed with 3.7% formaldehyde in a phosphate-buffered saline solution for 60 min under vacuum filtration. After fixation, cotyledons were stained with Hoechst 33342 (5 μg/ml; Life Technologies, H3570) for 4 hours, and then imaged using a confocal microscope.

### Computational models and simulations

The lineage decision tree model and all associated simulations were built and performed in MATLAB. The lineage decision tree model is a stochastic, asynchronous rule-based model of meristemoid progression through growth, asymmetric division, and differentiation. The starting sizes of 10,000 cells were randomly drawn from a gamma distribution (4, 33.3). Cells with random starting sizes below 40 μm^2^ (5%) were randomly redrawn. The cells then divided asymmetrically with a division asymmetry drawn from a beta distribution (6.8, 14.6) with a noise factor ± 0.05, each forming a smaller daughter cell or meristemoid and a larger daughter cell or SLGC. SLGCs were discarded, while the meristemoids differentiated with some probability based on their current size using the binomial CDF at (current size, 100, 0.32). For instance, a cell of 32 μm^2^ would have a 50% chance of differentiating (i.e., the transition size). If the cell did not differentiate, then it grew by 3% ± 0.005% per hour to the power of a random cell cycle length, with the cell cycle length drawn from another gamma distribution (10, 20). Calculating the growth to the power of cell cycle length allows for asynchronicity (individual cells are different ages). Parametric distributions were obtained through biological measurements. The fittings of the starting size of the meristemoid population, division asymmetry, and cell cycle length to gamma or beta distributions were conducted with the fitdist function from the fitdistrplus package (*58*). Cell sizes were rounded to the nearest integer square micrometers. To simulate lineage progression where differentiation is controlled by time (number of prior ACDs) rather than size (fig. S4A), we measured the probabilities of differentiation following 0, 1, 2, 3, or 4 ACDs from time-lapse data. These values were used to guide the behavior of simulated meristemoids, which were allowed to divide or differentiate regardless of birth size but according to the number of prior ACDs. Meristemoid behavior in *ctr1* mutants (Fig. 3C) was simulated with a mean starting cell size of 65% of that of wild type and a growth rate of 2% per hour, per empirical estimates. Meristemoid behavior in the tetraploid (Fig. 3C) was simulated with a mean starting cell size of 160% of that of the wild type and a transition size of 134% of that of the wild type, per empirical estimates.

### Statistical analysis

Sample sizes and statistical tests for each figure panel are described in table S1. All statistical analyses in this manuscript were performed in RStudio. Except when comparing transition sizes (see below), unpaired Mann-Whitney tests were conducted to compare the means of two groups using the compare_means function in the ggpubr package (*59*).

Logistic regression was conducted with the glm.fit function with a binomial model in R (*60*). Classification accuracy was estimated from separate training and test datasets. Briefly, fivefold cross-validation was used to split a dataset of *n* = 95 cells into pairs of training and test data. In each case, the training dataset was used to estimate the logistics with the glm.fit function. Then, for each cell in the test dataset, cell size at birth was used to compute a division probability according to the logistic, and a predicted behavior (i.e., division or differentiation) was assigned by binarizing the probability with a threshold of 0.5. Accuracy was calculated as the percentage of cells with correctly predicted behavior, and the average accuracy across all five cross-validations was reported. For all graphs, *P* values from the unpaired Mann-Whitney tests or logistic regression model were directly labeled on these graphs.

The transition size for division/differentiation was operationalized as the size at which 50% of cells differentiate. As this is conceptually equivalent to estimates of the LD_50_ (median lethal dose) value, the amount of a toxin that causes death in half of the subjects, we used the dose.p function from the R package MASS (*61*) to obtain point estimates and standard errors for the transition size. Comparisons between transition sizes were performed via two-sided *t* tests from the summary statistics produced by dose.p.
