# Supplementary material for: A cell size threshold triggers commitment to stomatal fate in Arabidopsis
Source: Sci Adv. 2023 Sep 20;9(38):eadf3497. doi: 10.1126/sciadv.adf3497 (PMC10881030; doi:10.1126/sciadv.adf3497)
Supplement: Supplementary file 1 — Figs. S1 to S8 Table S1 [file sciadv.adf3497_sm.pdf]

Science Advances
AAAS

Supplementary Materials for

A cell size threshold triggers commitment to stomatal fate in *Arabidopsis*

Yan Gong *et al.*

Corresponding author: Dominique C. Bergmann, bergmann@stanford.edu

Sci. Adv. **9**, eadf3497 (2023)
DOI: 10.1126/sciadv.adf3497

This PDF file includes:

Figs. S1 to S8
Table S1

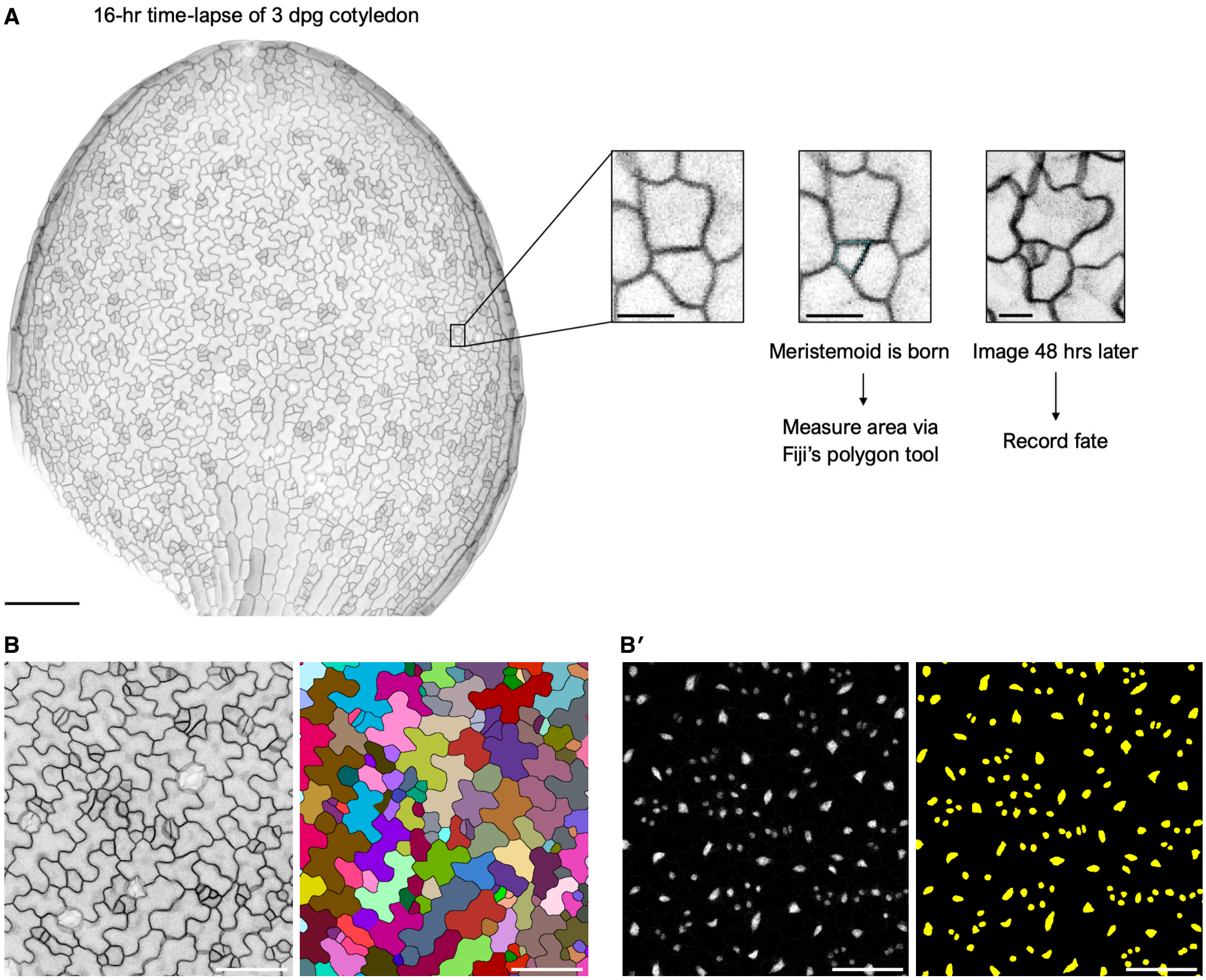

Fig. S1. General set-up for tracking cell sizes and fates over time.
(A) Schematic of experimental setup for timelapse analysis. Whole leaf images were acquired at 40 - 60 minute intervals over 16 hours to identify cells at birth and measure key features. Then, plants were allowed to recover for 48 hours and imaged once more to record fate outcomes such as further asymmetric division (shown) or differentiation into stomata. Typical cell cycle times in this tissue are approximately 12 - 16 hours. Scale bar: 100 µm, inset: 10 µm. (B-B') Examples of ilastik-based segmentation of cotyledon cell outlines (B, source on left, segmented cells on right) and cotyledon nuclei (B', source on left, segmented nuclei on right). Scale bar: 50 µm.

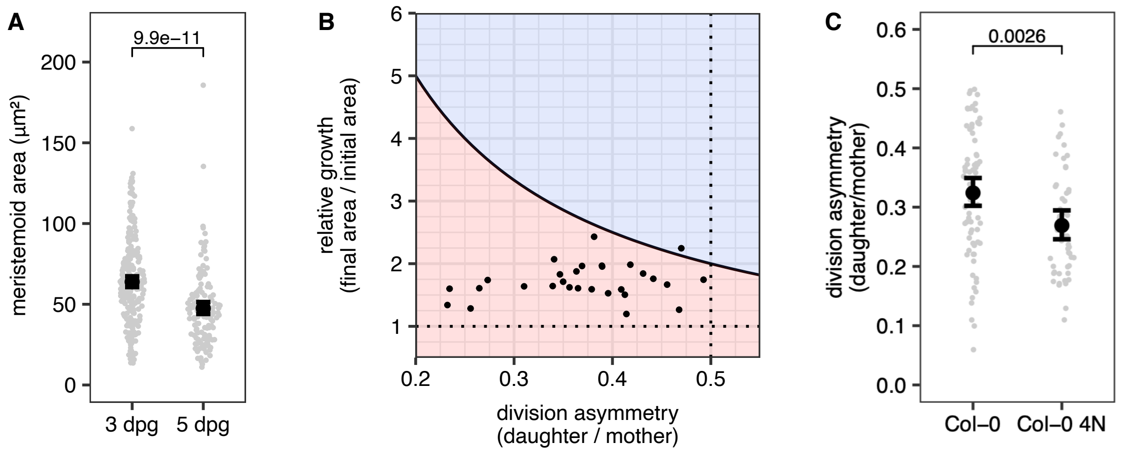

Fig. S2. Meristemoids decrease in size over successive divisions.
(A) Mean meristemoid area at 3- and 5- days post germination (dpg) shows a marked population-level decrease. (n>139 cells/age, p-value is calculated by Mann-Whitney test). (B) Tracking of individual meristemoids over the whole cell cycle (n = 25 cells). Cells typically grow to less than twice their birth size (y-axis) and divide with strong physical asymmetry (x-axis), which ensures daughter cells are smaller at birth than their mothers one cell cycle earlier. The curved line marks combinations of growth and asymmetry that maintain cell size (e.g. doubling in area with 50:50 division asymmetry). (C) Division asymmetry in Col-0 and Col-0 4N meristemoids (n > 50 cells/genotype).

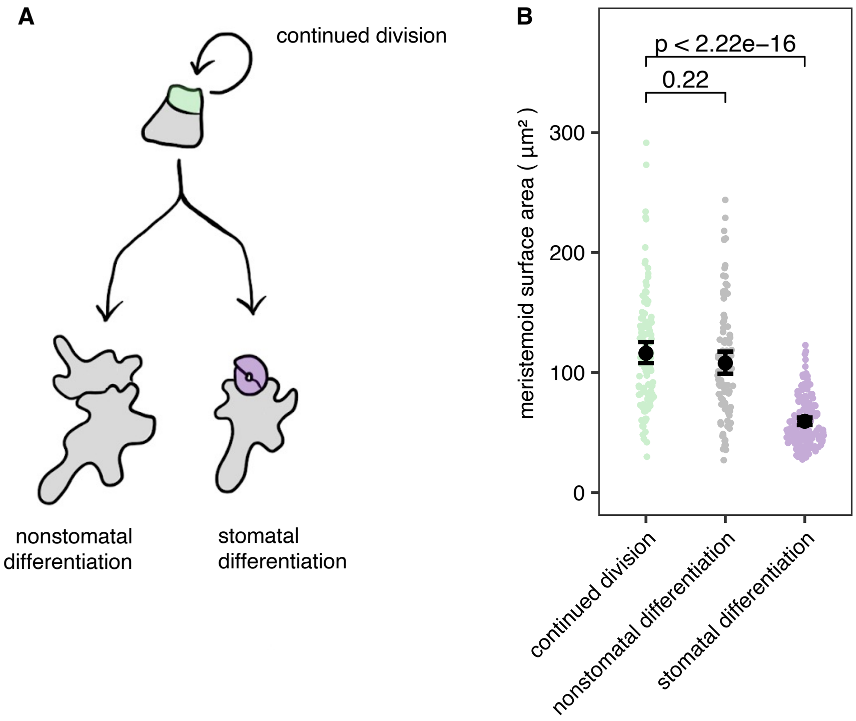

Fig. S3. Size-dependent differentiation is specific to the stomatal lineage.
(A) In tomato (*Solanum lycopersicum*), meristemoids can differentiate into either pavement cells or stomata (*28*). (B) Meristemoid size by cell behavior. Nonstomatal differentiation into pavement cells occurs at any size, but stomatal differentiation into GMCs is restricted to small meristemoids. (Data shown as mean ± 95% CI. n > 98 cells/behavior. p-values are calculated by Mann-Whitney test).

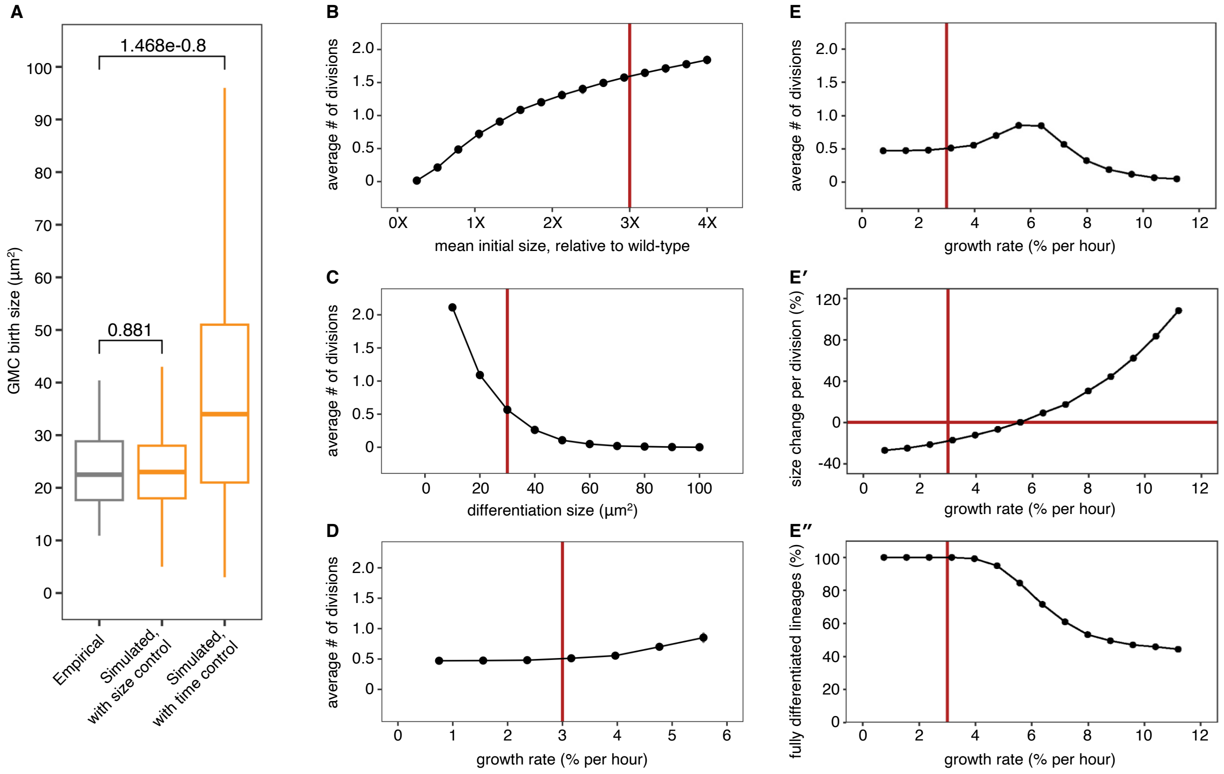

Fig. S4. Size-dependent differentiation integrates multiple possible inputs.
(A) Comparison of GMC birth sizes, measured from timelapse data or simulated using either cell size (size control) or the number of prior cell generations (time control) to guide differentiation. (B-E) Simulations of average number of amplifying divisions before differentiation as a function of inputs to the lineage model. (B) Initial size, in multiples of the average WT size. (C) Average size at differentiation, in μm^2. (D) Growth rate, in % per hour. (E-E'') Expanded analysis of growth rates, showing that above ~5% growth per hour, simulated cells switch from shrinking to growing with each division, and consequently many lineages do not differentiate within the simulation run (12 cell cycles). Vertical red lines indicate estimated WT values for each parameter. Black dots represent means of 10,000 simulated cell lineages and means of groups of 1000 cell lineages (simulated individuals) are shown in grey. p-values are calculated by the Kolmogorov-Smirnov test. Sample sizes: A: 50 real cells in 2 individuals, >7500 simulated cells.

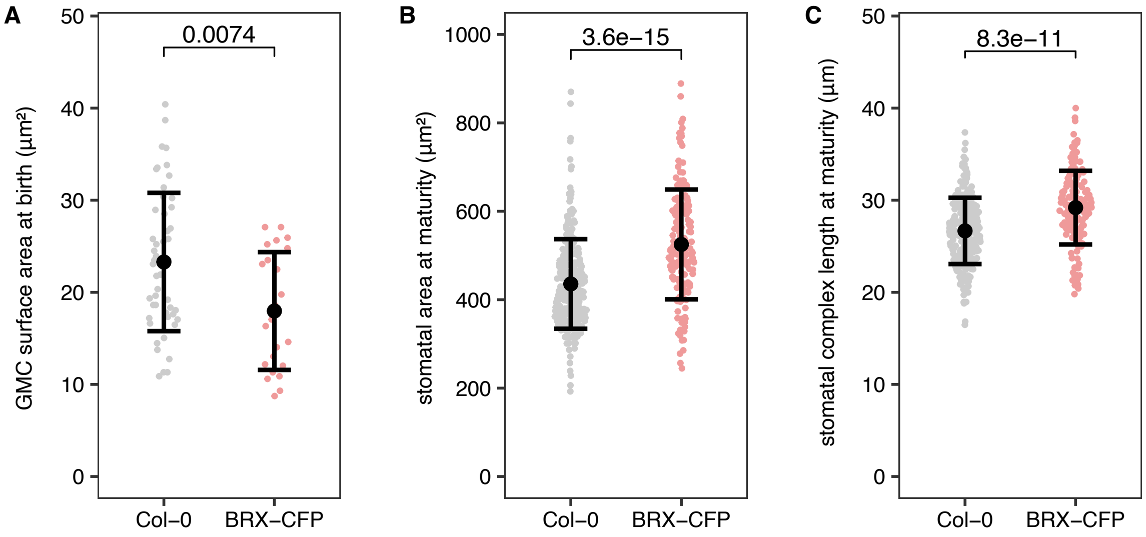

Fig. S5. The meristemoid transition cell size does not set the final size of stomata.
(A) GMC surface area at birth in Col-0 or a line ectopically expressing BRX-CFP (*pBASL::BRX-CFP)* (*50*) in the stomatal lineage (n>23 cells/genotype). (B) Surface area of mature stomatal complexes (both guard cells, measured at 21 dpg, n>169 stomata/genotype). (C) Length of mature stomatal complexes (measured at 21 dpg, n>169 stomata/genotype). All p-values are calculated by Mann-Whitney test.

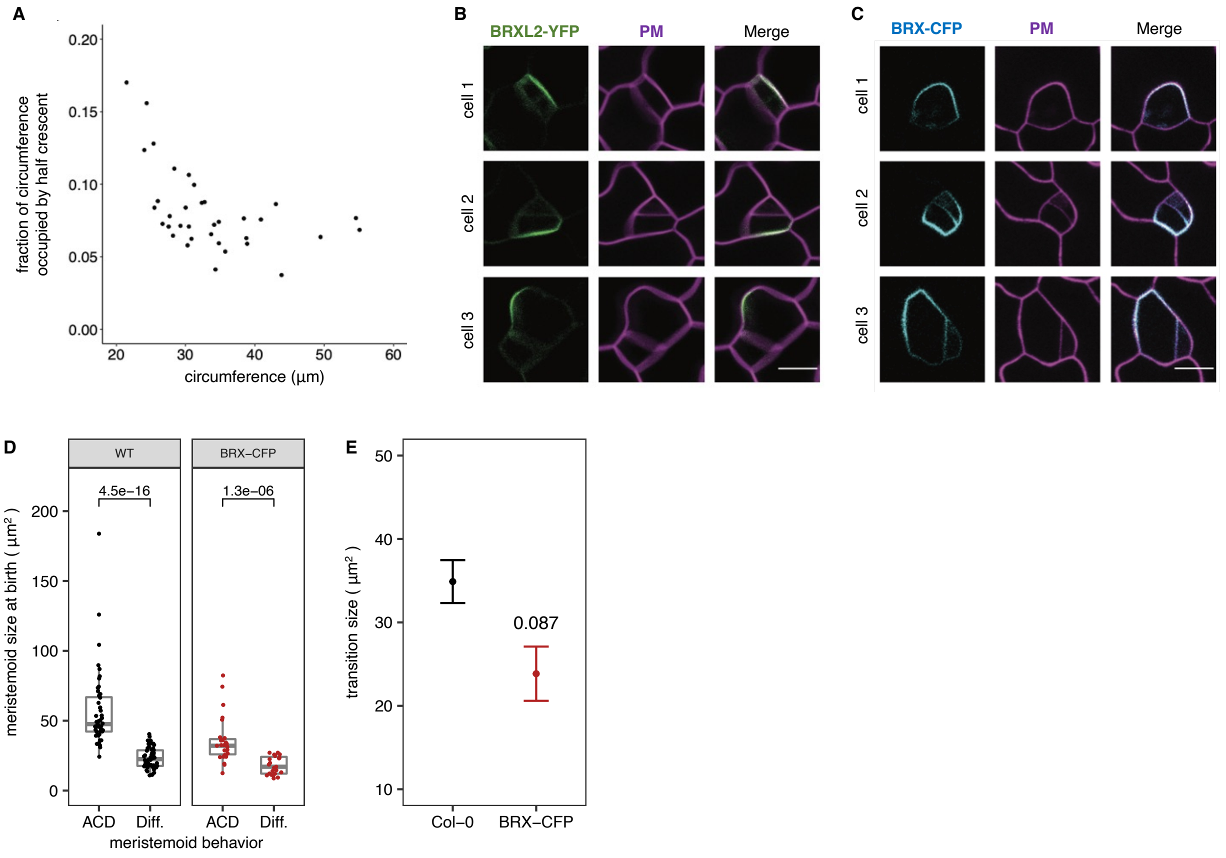

Fig. S6. Effects of polarity on size-dependent differentiation.

(A) Plot of the relationship between the relative proportion of plasma membrane that is occupied by BRXL2 polarity crescent (as standard deviation of the fitted curve divided by cell circumference) and the circumference of the cell (n = 35 cells). With the increase of the circumference, BRXL2 occupies a decreased proportion of the plasma membrane, indicating a lack of scaling for the BRXL2 polarity crescent. Both cell circumference and the SD of BRXL2 polar crescent are measured by POME V2.0 in FIJI. (B) Confocal images of BRXL2-YFP reporter in three representative stomatal lineage cells of different sizes. Cell 1 shows pre-divisional BRXL polarity and cells 2-3 show post-divisional BRXL2 polarity. *pBRXL2::BRXL2-YFP* (left), *pATML1::RCI2A-mCherry* (middle), and merged (right) are shown separately. (C) Confocal images of BRX-CFP reporter in three representative stomatal lineage cells of different sizes *pBASL::BRX-CFP* (left), propidium iodide staining (middle), and merged (right) are shown separately. Cell 1 shows pre-divisional BRXL polarity and cells 2-3 show post-divisional BRXL2 polarity. Note that in cells with particularly broad BRX crescents (e.g., Cell 2), the polar crescent can be bisected by the division plane and inherited by both daughter cells. (D) Comparison of cell size at birth between meristemoids that acquire different fates in Col-0, and the BRX-CFP reporter line (n> 50 cells/genotype). (E) Comparison of sizes at which meristemoids transition to predominantly differentiating (n> 50 cells/genotype, see methods). p-values are calculated by Mann-Whitney test in (D) and by a t-test on transition size estimates in (E). Scale bars, 10 µm.

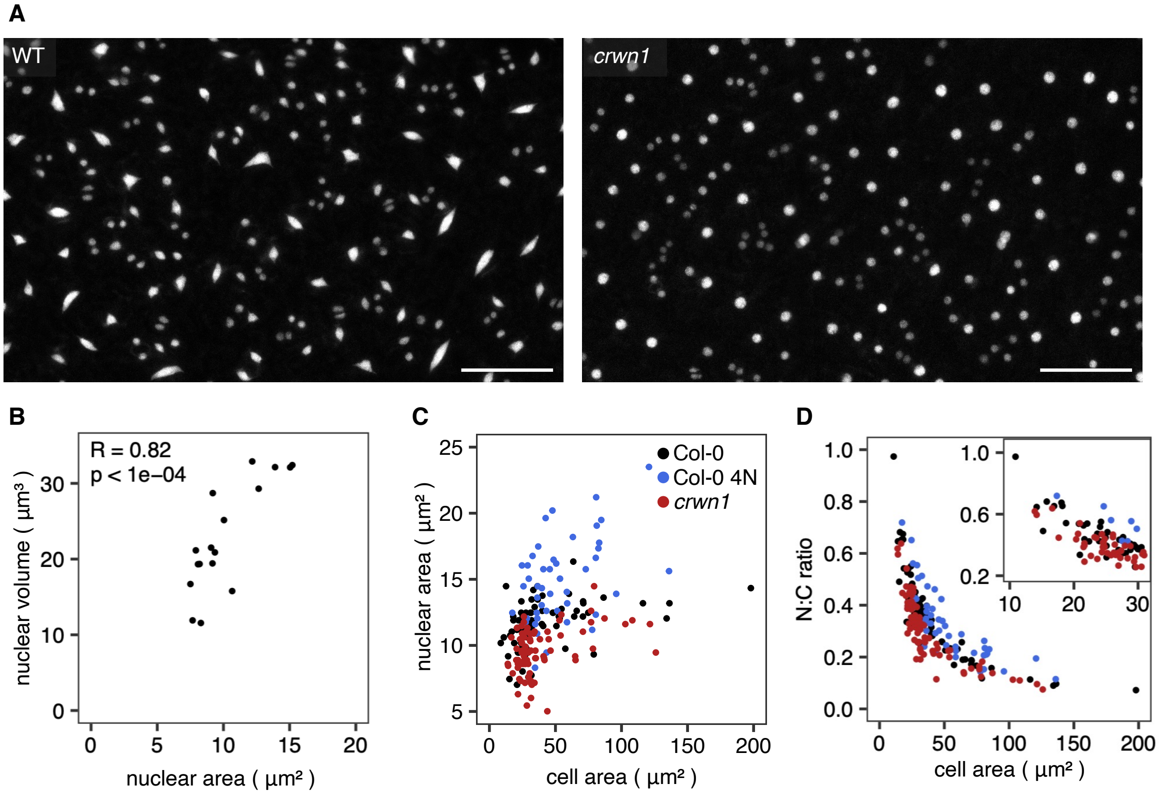

Fig. S7. *CRWN1* controls nuclear size without affecting overall cell size.
(A) Confocal images of WT and *crwn1* nuclei tagged with *pATML1::H2B-mTFP*. Scale bar: 50 µm. (B) Relationship between nuclear area and nuclear volume estimates in Col-0 (n = 17 cells). (C) Relationship between cell area and nuclear area (n>45 cells/genotype). (D) Relationship between cell area and N:C ratio (n>45 cells/genotype). Inset shows relationship for the smallest cells in the same dataset.

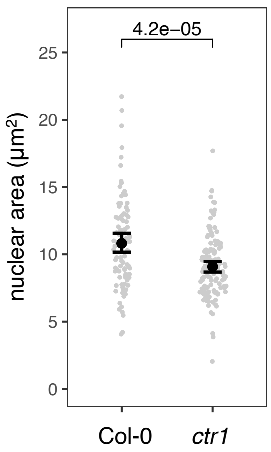

Fig. S8. Nuclear areas in Col-0 and *ctr1* meristemoids.
Comparison of nuclear areas in Col-0 and *ctr1* meristemoids in cotyledons at 3 dpg (n = 3 leaves/genotype and >90 nuclei/genotype), shown as mean and 95% confidence interval.

Figure panel	Description of data	Sample size	Error bars	Statistical test
1A	Schematic of stomatal development	N/A	N/A	N/A
1B	Example micrographs of meristemoids undergoing ACDs or differentiation	N/A	N/A	N/A
1C	Example micrographs of meristemoids undergoing ACDs or differentiation	N/A	N/A	N/A
1D	SPCH-YFP intensity at birth by cell behavior	ACD: 32 cells in 1 individual, differentiating: 16 cells in 1 individual	N/A	N/A
1E	SPCH-YFP intensity over time by cell behavior	ACD: 32 cells in 1 individual, differentiating: 16 cells in 1 individual	N/A	N/A
1F	MUTE-YFP intensity over time by cell behavior	ACD: not detected, differentiating: 5 cells in 1 individual	N/A	N/A
2A	Example micrographs of meristemoids undergoing ACDs or differentiation	N/A	N/A	N/A
2B	Cell size by cell behavior	ACD: 50 cells in 1 individual, differentiating: 50 cells in 1 individual	N/A	Mann-Whitney test
2C	Cell size by cell behavior	ACD: 50 cells in 1 individual, differentiating: 50 cells in 1 individual	N/A	N/A
3A	Cell size at birth	Empirical: 132 cells in 2 individuals	N/A	N/A
3A	Division asymmetry	Empirical: 98 cells in 2 individuals	N/A	N/A
3A	Cell size at birth by cell behavior	Empirical: 98 cells in 2 individuals	N/A	N/A
3A	Cell cycle length	Empirical: 112 cells in 2 individuals	N/A	N/A
3B	Rounds of amplifying division	Simulated: 913 cells	N/A	Mann-Whitney test
3C	Rounds of amplifying division	Empirical: Col-0: 988 cells across 3 individuals, Col-0 4N: 636 cells across 3 individuals, *ctr1*: 828 cells across 3 individuals. Simulated: in all cases, 10000 cells in each of 50 replicates. Data from Col-0 and *ctr1* taken from (*8*).	95% CI	N/A
4A	Epidermal cell sizes at 0 dpg	N/A	N/A	N/A
4B	Epidermal cell sizes at 0 dpg	Col-0: 541 cells, Col-0 4N: 486 cells, *ctr1*: 499 cells per each of 2 individuals	N/A	Mann-Whitney test
4C	Epidermal cell sizes at 4 dpg	Col-0: 63 cells, Col-0 4N: 49 cells, *ctr1*: 92 cells per each of 5 individuals	N/A	Mann-Whitney test
4D	Rounds of amplifying division	Col-0: 988 cells across 3 individuals, Col-0 4N: 636 cells across 3 individuals, *ctr1*: 828 cells across 3 individuals. Data from Col-0 and *ctr1* taken from (*8*).	N/A	N/A
4E	Rounds of amplifying division	Col-0: 988 cells across 3 individuals, Col-0 4N: 636 cells across 3 individuals, *ctr1*: 828 cells across 3 individuals. Data from Col-0 and *ctr1* taken from (*8*).	95% CI	Mann-Whitney test
4F	Cell sizes at birth by cell behavior	Col-0: 145 cells in 2 individuals, Col-0 4N 92 cells in 1 individual, *ctr1* 67 cells in 1 individual, *myoxi-i*: 87 cells in 1 individual	N/A	Mann-Whitney test

4G	Transition sizes across genotypes	Col-0: 145 cells in 2 individuals, Col-0 4N 92 cells in 1 individual, *ctr1* 67 cells in 1 individual, *myoxi-i*: 87 cells in 1 individual	95% CI	Two-sided t-test on transition size estimate
5A	Nuclear sizes at birth by cell behavior	Col-0: 77 cells in 1 individual, *crwn1*: 79 cells in 1 individual	N/A	Mann-Whitney test
5B	Cell sizes at birth by cell behavior	Col-0: 79 cells in 1 individual, *crwn1*: 80 cells in 1 individual	N/A	Mann-Whitney test
5C	Rounds of amplifying division	Col-0: 600 cells in 6 individuals, *crwn1*: 600 cells in 6 individuals	N/A	N/A
5D	Rounds of amplifying division	Col-0: 600 cells in 6 individuals, *crwn1*: 600 cells in 6 individuals	95% CI	Mann-Whitney test
5E	Cell sizes at transition	Col-0: 227 cells in 5 individuals, *crwn1:* 207 cells in 4 individuals	95% CI	Two-sided t-test on transition size estimate
5F	Nuclear sizes at transition	Col-0: 227 cells in 5 individuals, *crwn1:* 207 cells in 4 individuals	95% CI	Two-sided t-test on transition size estimate
5G	N:C ratios at transition	Col-0: 227 cells in 5 individuals, *crwn1:* 207 cells in 4 individuals	95% CI	Two-sided t-test on transition size estimate
5H	Nuclear sizes at transition	Col-0: 240 cells in 5 individuals, Col-0 4N: 100 cells in 3 individuals	95% CI	Two-sided t-test on transition size estimate
5H	Cell sizes at transition	Col-0: 240 cells in 5 individuals, Col-0 4N: 100 cells in 3 individuals	95% CI	Two-sided t-test on transition size estimate
S1A	Schematic of imaging strategy	N/A	N/A	N/A
S1B	Examples of cell and nuclear segmentation	N/A	N/A	N/A
S2A	Cell sizes by plant age	3 dpg: 241 cells in 1 individual, 5 dpg: 139 cells in 1 individual	95% CI	Mann-Whitney test
S2B	Relative growth and division asymmetry	25 cells in 1 individual	N/A	N/A
S2C	Division asymmetry in Col-0 and Col-0 4N	Col-0: 80 cells in 1 individual, Col-0 4N: 50 cells in 1 individual	95% CI	Mann-Whitney test
S3A	Schematic of stomatal development in tomato	N/A	N/A	N/A
S3B	Cell size by cell behavior	360 cells in 1 individual: 113 ACD, 98 NSD, 149 SD	95% CI	Mann-Whitney test
S4A	GMC sizes at birth	Empirical: 50 cells in 2 individuals, simulated: >7500 cells	N/A	Kolmogorov-Smirnov test
S4B	Simulation: rounds of amplifying division by initial size	Simulated: 10000 cells across 10 individuals	N/A	N/A
S4C	Simulation: rounds of amplifying division by growth rate	Simulated: 10000 cells across 10 individuals	N/A	N/A
S4D	Simulation: rounds of amplifying division by differentiation size	Simulated: 10000 cells across 10 individuals	N/A	N/A
S4E	Simulation: rounds of amplifying division by growth rate, expanded	Simulated: 10000 cells across 10 individuals	N/A	N/A
S5A	Cell size by genotype	Col-0: 50 cells in 1 individual, BRX-CFP: 23 cells in 1 individual	95% CI	Mann-Whitney test

S5B	Mature stomatal areas by genotype	Col-0: 268 stomata in 4 individuals, BRX-CFP: 169 stomata across 6 individuals	95% CI	Mann-Whitney test
S5C	Matural stomatal lengths by genotype	Col-0: 268 stomata in 4 individuals, BRX-CFP: 169 stomata across 6 individuals	95% CI	Mann-Whitney test
S6A	Crescent size by cell size	35 cells in 1 individual	N/A	N/A
S6B	Example micrographs of BRXL2-YFP crescents	N/A	N/A	N/A
S6C	Example micrographs of BRX-CFP crescents	N/A	N/A	N/A
S6D	Cell sizes at birth by cell behavior	Col-0: 98 cells in 2 individuals (same as Fig. 4F), BRX-CFP: 50 cells in 1 individual	N/A	Mann-Whitney test
S6E	Cell sizes at transition by genotype	Col-0: 98 cells in 2 individuals (same as Fig. 4F), BRX-CFP: 50 cells in 1 individual	95% CI	Two-sided t-test on transition size estimate
S7A	Example micrographs of Col-0 and *crwn1* nuclei	N/A	N/A	N/A
S7B	Nuclear volume vs. nuclear area	15 cells in 1 individual	N/A	N/A
S7C	Cell area vs. nuclear area	Col-0: 76 cells in 1 individual, Col-0 4N: 45 cells in 1 individual, *crwn1*: 79 cells in 1 individual	N/A	N/A
S7D	Cell area vs. N:C ratio	Col-0: 74 cells in 1 individual, Col-0 4N: 45 cells in 1 individual, *crwn1*: 79 cells in 1 individual	N/A	N/A
S8	Nuclear areas in Col-0 and *ctr1*	Col-0: 92 cells in 3 individuals, *ctr1*: 139 cells in 3 individuals	95% CI	Mann-Whitney test

Table S1. Summary of sample sizes and statistical tests for all figures.
